# Editorial: Advances in brain disorders: from mechanisms to therapeutic targets

**DOI:** 10.3389/fnmol.2023.1337583

**Published:** 2023-11-28

**Authors:** Federica Bono, Giulia Abate, Sara Anna Bonini, Maria Josè Sisalli, Arianna Bellucci

**Affiliations:** ^1^Department of Molecular and Translational Medicine, University of Brescia, Brescia, Italy; ^2^Division of Pharmacology, Department of Neuroscience, School of Medicine, University of Naples “Federico II”, Naples, Italy

**Keywords:** brain disorders, brain disease mechanisms, disease biomarker discovery, therapeutic targets, therapeutic development

Brain disorders represent one of the main challenges for global society because of their outstanding impact on human health and social welfare (Feigin et al., 2020; Arias et al., 2022). This is aggravated by the detrimental effects of COVID-19 infections on the central nervous system and more generally on human wellbeing, which predispose a large number of affected patients to the development of acute or chronic neurological disorders or mental illnesses (Heneka et al., 2020; Penninx et al., 2022).

More efficient strategies to monitor brain health are warranted in order to precociously diagnose neurological and psychiatric disorders and finely stage their progression. Still, the development of novel therapeutic approaches able to halt disease advancement without inducing severe side effects is compelling in order to factually cure patients affected by brain disorders. Such objectives can be reached only by the elucidation of the key molecular underpinnings of brain disorders and by the combination and integration of data deriving from basic and clinical research studies.

This Frontiers in Molecular Neuroscience Topic collects research articles and reviews providing novel significant advancements in our general knowledge of the biological basis of disease and opening new insights into the development of innovative biomarkers and therapeutic approaches for these disabling conditions.

Guo et al. showed that Rab20 inhibition significantly alleviates brain infarct volume, neurological deficits, and apoptosis by inhibiting mitochondrial fission and dysfunction in models of ischemia/reperfusion injury.

McClendon et al. showed that the administration of the steroid receptor co-activator MCB-10-1 in a rat model of transient cerebral ischemia reduces post-ischemic brain damage by modulating microglia and astroglia and mitigating neurologic impairment.

Shen et al. reviewed the contribution of single-cell sequencing technology to our understanding of post-stroke brain damage.

Wang et al. summarized the role of chemokines in intracerebral hemorrhage, underlining their key role as disease mediators and highlighting their relevance as therapeutic targets.

Fan G. et al. reviewed the role of glutamate in excitotoxicity and ferroptosis in the context of ischemic stroke, describing possible therapeutic approaches to modulate these events.

Shim et al. investigated the occurrence of shared pathological pathways between cerebral adrenoleukodystrophy and Alzheimer's disease through an *in silico* approach, showing that annexin A5, beta-2-microglobulin, CD44, and fibroblast growth factor 2 associate with the pathogenesis of the two neurodegenerative diseases.

Guan et al. investigated the pathogenic mechanism of abnormal cell cycle re-entry of neurons in Alzheimer's disease in *in vitro* and *in vivo* models with the β-amyloid pathology, revealing that increased expression of cyclin-dependent kinase (CDK)1/2/4 and cyclin A2/B1/D3/E1, and a parallel decrease of p18 and p21 underlie β-amyloid protein-dependent-enhanced cell cycle re-entry, which can be counteracted by aspirin administration.

Gasser et al. showed that a microglial/innate immune modulator selectively targeting microglial aberrant functions, such as synaptic overpruning, may attenuate the progression of cognitive, and motor decline in Huntington's disease.

Brembati et al. reviewed the significance of post-translational modifications in α-synuclein, a protein playing a major role in the pathophysiology of Parkinson's disease, opening insightful perspectives for the development of innovative therapeutic approaches or disease biomarkers.

In their minireview, Zhao et al. described the relevance of the vesicular glutamate transporters to Parkinson's disease pathophysiology.

Mays et al. found that plasmin inhibits the formation of pathological misfolded prion protein scrapie by mediating prion protein α-cleavage that, in turn, reduces prion conversion.

Lu et al. identified a *de novo* nonsense mutation of FOXG1 in a Chinese patient affected by neurodevelopmental disorders/intellectual disorders (NDDs/IDs) and clarified the diagnostic criteria defining the FOXG1-related clinical phenotypes.

By assessing the efficacy of dodecyl creatine ester in the Slc6a8 knockout mouse model through shotgun proteomics on brain proteins, Mabondzo et al. found that phospholipase C beta 1, kinesin family member 1A, and associated molecules are crucially involved in the pathogenesis of creatine transporter deficiency, a leading cause of intellectual disability.

Shao et al. showed that the severely impaired cognitive subtype in the early course of schizophrenia is characterized by differences in the brain's spontaneous neural activity of the prefrontal cortex and bilateral posterior cingulate cortex/precuneus.

Peng et al. described a case report of a patient diagnosed with Wiedemann–Rautenstrauch syndrome (WDRTS) complicated with the occurrence of another recessive disorder, Fanconi anemia (FA).

Zhang Z. et al. characterized plasma metabolic profiles associated with brain atrophy in individuals with alcohol dependence, reporting that glycerophospholipid metabolism may have a prominent role in the pathogenesis of alcohol-induced brain atrophy.

Nie et al. reviewed the role and the current studies of pyroptosis, ferroptosis, parthanatos, and cyclophilin D-mediated necrosis in traumatic brain injury secondary damage, discussing whether these signaling pathways may provide new insights into the treatment of craniocerebral injury.

In their review, Tian et al. discussed the regulatory mechanisms of cyclin-dependent kinase 5 (Cdk5) in a series of common neurological disorders such as neurodegenerative diseases, stroke, anxiety/depression, pathological pain, and epilepsy, emphasizing the crucial role of Cdk5 aberrant activation as a driving force for the initiation and progression of brain injury.

In their review, Fan Y. et al. summarized the most recent developments on the role of miRNAs in the regulation of reactive astrocytes in CNS diseases, elegantly discussing the clinical application of miRNA-based therapies for the modulation of reactive astrocytosis.

Finally, Zhang Y. et al. investigated the cellular and molecular mechanisms related to primary familial brain calcification by using Slc20a2 homozygous knockout mice, showing that impairment in the paracellular and transcellular pathways of the brain endothelial cells produces BBB leakage and brain T-cell invasion that, in turn, underlie the onset of brain calcification.

## Author contributions

FB: Writing – original draft, Writing – review & editing. GA: Writing – original draft, Writing – review & editing. SB: Writing – original draft, Writing – review & editing. MS: Writing – original draft, Writing – review & editing. AB: Writing – original draft, Writing – review & editing.
